# Psychological impact of visible differences in patients with congenital craniofacial anomalies

**DOI:** 10.1186/s40510-015-0078-9

**Published:** 2015-04-18

**Authors:** Varun Pratap Singh, Timothy P Moss

**Affiliations:** Department of Orthodontics and Dentofacial Orthopedics, Nobel Medical College and Teaching Hospital, Kanchanbari, Biratnagar 56700 Nepal; Department of Health Psychology, University of the West of England, Frenchary Campus, cold Harbour Lane, Bristol, BS161QY UK

**Keywords:** Craniofacial anomalies, Dental aesthetics, Facial aesthetics, Orthodontic, Psychosocial, Derriford

## Abstract

**Background:**

Patients with craniofacial anomalies often have appearance concerns and related social anxiety which can affect their quality of life. This study assessed the psychological impact of facial and dental appearance in patients with craniofacial anomalies in comparison to a general population control group.

**Methods:**

The study involved 102 adult patients (51% male) with congenital craniofacial anomalies and 102 controls (49% male). Both groups completed the Nepali version of Derriford Appearance Scale (DAS) and the Psychological Impact of Dental Aesthetic Questionnaire (PIDAQ) in a clinical setting to assess appearance-related distress, avoidance, and anxiety.

**Results:**

There was a significant difference between patients and controls on both PIDAQ (mean score for patients 33.25 ± 9.45 while for controls 27.52 ± 5.67, *p* < 0.001) and DAS59 scores (mean score for patients 159.16 ± 31.54 while for controls 77.64 ± 6.57, *p* < 0.001), indicating that patients experienced greater negative psychological impact of living with their appearance (PIDAQ) and more appearance-related distress (DAS) than controls. DAS scores were not associated with gender. There was no association of the place of residence (rural vs. urban) with PIDAQ or DAS59 scores.

**Conclusions:**

There is a significant psychological impact of altered facial and dental appearance in patients with craniofacial anomalies compared to controls. There was no effect of locality (rural/urban) on the psychological impact of facial and dental appearance in patients.

## Background

Craniofacial anomalies can be defined as the conditions that encompass all congenital deformities of the cranium and face. More specifically, however, the term has come to imply congenital deformities of the head and face that interfere with physical and mental well-being [1].

The spectrum of craniofacial anomalies is very diverse, and the most common conditions include (but not restricted to) cleft lip and/or palate, craniosynostosis (which may be associated with Crouzon’s syndrome or Apert’s syndrome), otomandibular anomalies (Treacher Collins syndrome), CHARGE associations, holoprosencephaly, Stickler syndrome, and fetal alcohol syndrome [2,3]. The clinical features include a spectrum of deformities of the craniofacial region including cranium and cranial sutures, and deformity of skull shape and facial bones including the maxilla, mandible, zygomatic arches, nose, eyes, ears, lips, and teeth [4-10].

Patients with abnormal facial appearance often have to face social discrimination. Individuals with abnormal facial appearance are typically considered to be less attractive and are often stereotypically considered as less capable, less intelligent, and less honest. Their facial appearance interferes with personal life, employability, and social interaction. Many investigations have shown that these disfiguring conditions can lead to various psychosocial problems such as high level of social anxiety and social avoidance, and poorer quality of life [11-13].

The potential problems in this patient group are further compounded by an increased prevalence of dental anomalies and malocclusion [14-18]. The best treatment approach is multidisciplinary, which includes teamwork and support from various specialties. During almost all phases of treatment, dental services are needed and orthodontists are almost always needed from early treatment until late adult life [19]. Good facial and dental aesthetics may have a beneficial role on behavior and self-esteem. Clinicians are expected to produce evidence of the quality of care they deliver. To this end, it is necessary to use standardized, valid, and reliable psychological as well as clinical measures to evaluate outcomes [20-22].

The research in this area is somewhat conflicting and suffers various lacunae for methodological reasons. These include inconsistency in psychometric scales and constructs used, the lack of validity and reliability in many of the measures, small sample sizes, and no sub-categorization of anomalies among other methodological errors [23]. Investigators have highlighted the need to move towards a ‘social science model’ from a ‘medical model’ [24-27].

This study was undertaken to assess the impact of psychological adjustment due to altered facial and dental appearance in patients with craniofacial anomalies utilizing the Derriford Appearance Scale [28] and Psychological Impact of Dental Aesthetic Questionnaire (PIDAQ) [20] which are specially designed for evaluating the psychological adjustment in people with visible differences in appearance. There are few such studies utilizing the Derriford Appearance Scale [29] and no study studying the psychological impact of facial and dental aesthetics together.

There is a strong *prima facie* case for comparing rural and urban populations with regard to appearance satisfaction in Nepal. There are known differences in healthcare access, service utilization, and geographic distribution of providers and services in healthcare in Nepal, with rural communities typically experiencing greater difficulties than urban comparators [30]. Given the potential for increased social isolation on one hand, but possibility of reduced prevalence of contemporary appearance pressures in more isolated communities on the other, and the likely relation between this and appearance expectations and outcomes, we included an exploratory investigation of rural versus urban populations within this study.

The objectives of this study were therefore:

1. To assess the psychological impact of facial and dental appearance in patients with craniofacial anomalies in comparison to a general population sample.
2. To explore the relationship between urban and rural residence in relation to psychosocial impact of facial and dental anomalies.

The following hypotheses were put forward:

- H1 - There is a psychosocial impact of facial and dental appearance on patients with craniofacial anomalies.
- H2 - There is a difference in psychosocial impact of facial and dental appearance of rural and urban patients.

## Methods

This study was conducted in two steps. The first step included translation and validation of the instruments - PIDAQ and DAS59 in the target population. This part is described in detail as published data elsewhere [31,32]. The second step included assessment of patients with craniofacial anomalies reporting for orthodontic treatment using these validated instruments.

The study was conducted in the Department of Orthodontics, BP Koirala Institute of Health Sciences, Dharan, Nepal, from 1 February 2011 to 30 October 2012. Ethical clearance was obtained from Institutional Ethical Committee, B.P. Koirala Institute of Health Sciences, Dharan, Nepal, reference no. Acd/216/068/069, and principles from the Declaration of Helsinki were followed. The study population consisted of adult patients with congenital craniofacial anomalies visiting the Department of Orthodontics during the above said period. The study also included similar patients who were referred/or reported to the orthodontic OPD during 2005 to 2010 and did not undergo orthodontic treatment and patients from the waiting list for whom treatment had not started. The inclusion criteria were adult patients with congenital craniofacial anomalies aged 18 to 30 years. Patients with acquired or traumatic facial disfigurement and history of orthodontic treatment, people who did not have the capacity to offer informed consent, and people who could not read the test booklet unaided were excluded.

There were 112 patients satisfying the inclusion and exclusion criteria. All were invited to participate, and 102 (91%) agreed to participate in the study. Written informed consent was obtained from all the participants. There were 52 males and 50 females with a mean age of 24.78 years (s.d. = 2.5). Forty-six were from rural areas and 56 from urban. Details regarding the classification of patients according to the craniofacial anomalies are presented in Table 1. A similar number of controls, 50 males and 52 females (mean age 24.99 years, s.d. = 2.73), were selected as a convenience sample from the university students and employees who have no acquired or congenital facial deformity. Thirty-nine were from rural areas and 63 from urban. Participants with severe malocclusion as assessed by an orthodontist were not included. The exclusion criteria were the same as those of the case groups.

**Table 1:** **Classification of patients who participated in the study according to the diagnosis of craniofacial anomalies**

**Serial number**	**Craniofacial anomalies**	**Number (102)**
1.	Isolated cleft lip/palate	45
2.	Isolated craniosynostosis	22
3.	Hemifacial microsomia	10
4.	Ectodermal dysplasia	4
5.	Cleidocranial dysplasia	3
6.	Treacher Collins syndrome	6
7.	Pierre Robin syndrome	4
8.	Crouzon’s syndrome	4
9.	Apert’s syndrome	4

The questionnaire pack consisted of

1. An introductory section with basic demographic information including age, sex, and place of residence in terms of rural and urban.
2. Nepali version of DAS consisting of 59 items. Each item response is marked based on a Likert scale from 1 to 4, with 1 indicating ‘almost never’ and 4 indicating ‘almost always’.
3. Nepali version of PIDAQ consisting of 23 items arranged in four domains. Each item response is marked based on a Likert scale from 0 to 4, with 0 indicating ‘not at all’ and 4 indicating ‘very strongly.’

These questionnaires were administered to the participants by one author who is well trained in this procedure. The patients were seated in a private room, in the Department of Orthodontics, and were asked to fill the questionnaire pack without the aid of the investigators, minimizing the likelihood of demand characteristics biasing responses. Participants were paid legitimate expenses incurred in attending the session but otherwise offered no incentive to participate. Payment was entirely independent of participants’ responses and, as such, unlikely to induce any bias in responding. Furthermore, the level of remuneration was such that participants did not profit from participation and such were not induced to a particular response set.

### Statistical procedures

Descriptive statistics was calculated for the demographic data. Independent *t* tests were used to evaluate the differences between cases and controls for scores of DAS59 and PIDAQ scales. Independent *t* tests were used to assess the effect of gender (male vs. female) and locality (rural vs. urban) on PIDAQ and DAS59 scores in both cases and controls. Bonferroni corrections were applied to the results to correct for type 1 errors resulting from multiple comparisons.

## Results

There was a significant difference between patients and controls on PIDAQ (mean score for patients = 33.25, s.d. = 9.45; mean for controls = 27.52, s.d. = 5.67; *p* < 0.001) and DAS59 scores (mean score for patients = 159.16, s.d. = 31.54; mean for controls = 77.64, s.d. = 6.57; *p* < 0.001) (Table 2). The patients’ scores were significantly higher than those of controls on both the PIDAQ and DAS59 scales.

**Table 2:** **Differences between the DAS 59 and PIDAQ scores for patients and controls**

**Items**	**Group**	**Mean**	**Standard deviation**	***p*** **value**
General self-consciousness	Cases	49.29***	10.8	<0.001
	Controls	21.84***	3.2	
Social self-consciousness	Cases	58.23***	14.17	<0.001
	Controls	22.8***	2.40	
Sexual and bodily self-consciousness	Cases	24.26***	5.98	<0.001
	Controls	8.77***	2.09	
Negative self-concept	Cases	9.65***	3.16	<0.001
	Controls	16.91***	1.17	
Facial self-consciousness	Cases	12.02***	2.50	<0.001
	Controls	4.66***	1.06	
Physical	Cases	5.71***	1.60	<0.001
	Controls	2.65***	0.99	
Total DAS score	Cases	159.16***	31.54	<0.001
	Controls	77.64***	6.57	
Dental self-confidence	Cases	12.84***	3.10	<0.001
	Controls	11.11***	2.91	
Social impact	Cases	7.2**	3.36	0.004
	Controls	6.1**	1.72	
Psychological impact	Cases	6.33***	2.86	<0.001
	Controls	5.03***	1.92	
Aesthetic concern	Cases	6.88***	2.76	<0.001
	Controls	5.28***	1.62	
Dental self-consciousness	Cases	8.56***	4.20	<0.001
	Controls	6.65***	1.99	
Total PIDAQ score	Cases	33.25***	9.45	<0.001
	Controls	27.52***	5.67	

* correlation is significant at the 0.05 level.** correlation is significant at the 0.01 level.***correlation is significant at the 0.001 level.

### Gender

There was no effect of gender except for sub-domains of PIDAQ ‘psychological impact’ and ‘aesthetic concern,’ where females had higher scores than males (mean, M = 5.44 (s.d. = 2.2), F = 7.26 (s.d. = 3.18), *p* = 0.001; M = 4.27 (s.d. = 1.99), F = 5.32 (s.d. = 2.55), *p* =0.005). However, the total PIDAQ score was significantly higher in females as compared to males (mean, M = 31.25 (s.d. = 7.96), F = 35.34 (s.d. = 10.45), *p* = 0.02). DAS scores were not differentiated by gender (Table 3). In controls, there was no difference in PIDAQ and DAS59 scores by gender (Table 4).

**Table 3:** **Effect of gender on PIDAQ and DAS59 scores in patients**

**Domain**	**Sex**	**Mean**	**Standard deviation**	**Standard error mean**	***p*** **value**
General self-consciousness	Male	49.29	10.815	1.5	0.996
	Female	49.3	10.998	1.555	
Social self-consciousness	Male	58.73	14.096	1.955	0.715
	Female	57.7	14.379	2.033	
Sexual and bodily self-consciousness	Male	24.44	5.992	0.831	0.762
	Female	24.08	6.037	0.854	
Negative self-concept	Male	9.52	3.032	0.421	0.68
	Female	9.78	3.328	0.471	
Facial self-consciousness	Male	12.23	2.51	0.348	0.388
	Female	11.8	2.507	0.355	
Physical	Male	5.85	1.638	0.227	0.372
	Female	5.56	1.58	0.223	
Total DAS	Male	160.06	31.585	4.38	0.77
	Female	158.22	31.799	4.497	
Dental self-confidence	Male	12.69	2.86	0.397	0.62
	Female	13	3.369	0.476	
Social impact	Male	6.98	3.006	0.417	0.512
	Female	7.42	3.709	0.525	
Psychological impact	Male	5.44***	2.2	0.305	0.001
	Female	7.26***	3.18	0.45	
Aesthetic concern	Male	6.13**	2.385	0.331	0.005
	Female	7.66**	2.939	0.416	
Dental self-consciousness	Male	8.17	4.218	0.585	0.348
	Female	8.96	4.204	0.595	
Total PIDAQ score	Male	31.25*	7.963	1.104	0.028
	Female	35.34*	10.458	1.479	

* correlation is significant at the 0.05 level.** correlation is significant at the 0.01 level.***correlation is significant at the 0.001 level.

**Table 4:** **Effect of gender on PIDAQ and DAS59 scores in controls**

**Domain**	**Sex**	**Mean**	**Standard deviation**	**Standard error mean**	***p*** **value**
General self-consciousness	Male	21.84	3.046	0.431	0.992
	Female	21.85	3.438	0.477	
Social self-consciousness	Male	22.7	2.667	0.377	0.67
	Female	22.9	2.135	0.296	
Sexual and bodily self-consciousness	Male	8.92	2.311	0.327	0.494
	Female	8.63	1.869	0.259	
Negative self-concept	Male	16.98	1.22	0.173	0.566
	Female	16.85	1.127	0.156	
Facial self-consciousness	Male	4.56	0.812	0.115	0.371
	Female	4.75	1.266	0.176	
Physical	Male	2.58	0.883	0.125	0.506
	Female	2.71	1.091	0.151	
Total DAS	Male	77.58	6.843	0.968	0.932
	Female	77.69	6.379	0.885	
Dental self-confidence	Male	11.18	2.833	0.401	0.808
	Female	11.04	3.016	0.418	
Social impact	Male	6	1.702	0.241	0.575
	Female	6.19	1.749	0.243	
Psychological impact	Male	5.22	1.93	0.273	0.328
	Female	4.85	1.914	0.265	
Aesthetic concern	Male	5.5	1.681	0.238	0.19
	Female	5.08	1.557	0.216	
Dental self-consciousness	Male	6.84	1.707	0.241	0.34
	Female	6.46	2.236	0.31	
Total PIDAQ score	Male	27.9	5.319	0.752	0.509
	Female	27.15	6.024	0.835	

### Rural versus urban

There was no difference in the place of residence (rural vs. urban) with either PIDAQ or DAS59 scores in patients (Tables 5 and 6), nor for subscales of each of these measures following Bonferroni control for multiple comparisons.

**Table 5:** **Effect of locality (rural/urban) on PIDAQ and DAS59 scores in patients**

**Domain**	**Rural/urban**	**Mean**	**Standard deviation**	**Standard error mean**	**Significance**
General self-consciousness	Rural	49.91	10.167	1.499	0.604
	Urban	48.79	11.447	1.53	
Social self-consciousness	Rural	59.07	13.401	1.976	0.59
	Urban	57.54	14.864	1.986	
Sexual and bodily self-consciousness	Rural	24.63	5.867	0.865	0.579
	Urban	23.96	6.12	0.818	
Negative self-concept	Rural	9.59	2.941	0.434	0.863
	Urban	9.7	3.368	0.45	
Facial self-consciousness	Rural	11.76	2.349	0.346	0.347
	Urban	12.23	2.628	0.351	
Physical	Rural	5.89	1.464	0.216	0.293
	Urban	5.55	1.715	0.229	
Total DAS	Rural	160.85	29.731	4.384	0.626
	Urban	157.77	33.165	4.432	
Dental self-confidence	Rural	12.7	3.595	0.53	0.666
	Urban	12.96	2.669	0.357	
Social impact	Rural	7.37	3.555	0.524	0.639
	Urban	7.05	3.216	0.43	
Psychological impact	Rural	6.13	2.841	0.419	0.519
	Urban	6.5	2.892	0.386	
Aesthetic concern	Rural	6.85	3.048	0.449	0.91
	Urban	6.91	2.539	0.339	
Dental self-consciousness	Rural	9.09	4.56	0.672	0.253
	Urban	8.13	3.885	0.519	
Total PIDAQ score	Rural	33.04	10.321	1.522	0.839
	Urban	33.43	8.761	1.171	

## Discussion

In comparison to general population controls, craniofacial patients with orthodontic and orthognathic concerns showed greater appearance-related distress, according to valid and reliable psychometric scales. For general distress, there was also no difference between urban and rural participants. Females did not demonstrate more distress than males when assessed using the DAS59. However, on two of the dental-specific scales of the PIDAQ, ‘psychological impact’ and ‘aesthetic concern,’ female patients reported more distress than male patients.

Some studies and reviews suggest that there are few significant differences in overall psychological functioning of patients with craniofacial anomalies as compared to general population norms. However, these studies report some difficulty in a particular area of functioning [33-39]. There is substantial evidence of appearance concern due to dissatisfaction with facial appearance in patients with craniofacial anomalies [40-44]. This dissatisfaction with facial appearance may lead to behavioral difficulties [45-48].

Many studies point out that the adult population is at risk of psychosocial problems due to concerns regarding their facial appearance [49-52]. The results of this study have supported the hypothesis that adults who have craniofacial anomalies have negative psychosocial impact due to facial and dental appearance. In the current study, both DAS and PIDAQ scores were significantly higher in patients than in controls, indicating that patients experienced negative psychological impact and more distress (Table 2).

In this study, it was shown that there were no significant differences in DAS59 scores for male versus female patients. This is in accordance with Kiyak and Bell [53], who stated that there were no gender differences on psychological variables in the pre-surgical assessment of patients needing orthognathic surgery, though counter to other evidence regarding self-consciousness of appearance, which typically shows more distress among female participants [28]. In the analysis of the PIDAQ, it was interesting that there was statistically significant difference for overall psychological impact of dental aesthetics on two PIDAQ subscales, though when a Bonferroni correction for multiple comparisons is applied to the data, the difference becomes non-significant. However, the specific psychological impact upon males and females assessed using PIDAQ subscales does significantly differ, with females having higher scores for both (Table 3). It may be hypothesized that females are socialized to be more concerned and dissatisfied by their dental appearance as compared to males and to socially evaluate their appearance to a greater degree than males [54]. While it remains to be further investigated in subsequent research, it is possible that patients with craniofacial conditions are more sensitized to appearance distress than controls, and in combination with social pressure associated with gender, more distress ensues.

Contrary to our expectations, there was no effect of area of residence (rural vs. urban) on the overall psychological impact of facial and dental appearance in patients or control participants following Bonferroni correction for type 1 errors. This may indicate that aesthetic norms and pressures are not decreased by living in a less populous urban environment and that these norms are pervasive. It also suggests that the availability of complex cosmetic dentistry in urban Nepal has not fundamentally shifted the discrepancy between perceived dental appearance ideals and actuality.

**Table 6:** **Effect of locality (rural/urban) on PIDAQ and DAS59 scores in controls**

**Domain**	**Rural/urban**	**Mean**	**Standard deviation**	**Standard error mean**	**Significance**
General self-consciousness	Rural	21.46	2.584	0.414	0.351
	Urban	22.08	3.58	0.451	
Social self-consciousness	Rural	22.92	2.559	0.41	0.695
	Urban	22.73	2.315	0.292	
Sexual and bodily self-consciousness	Rural	8.33	2.144	0.343	0.094
	Urban	9.05	2.027	0.255	
Negative self-concept	Rural	16.62*	1.206	0.193	0.044
	Urban	17.1*	1.118	0.141	
Facial self-consciousness	Rural	4.49	0.823	0.132	0.208
	Urban	4.76	1.187	0.15	
Physical	Rural	2.69	0.977	0.157	0.719
	Urban	2.62	1.007	0.127	
Total DAS	Rural	76.51	6.476	1.037	0.176
	Urban	78.33	6.594	0.831	
Dental self-confidence	Rural	11.31	3.262	0.522	0.588
	Urban	10.98	2.697	0.34	
Social impact	Rural	5.82	1.819	0.291	0.201
	Urban	6.27	1.648	0.208	
Psychological impact	Rural	5.44	1.917	0.307	0.093
	Urban	4.78	1.896	0.239	
Aesthetic concern	Rural	5.72*	1.701	0.272	0.033
	Urban	5.02*	1.529	0.193	
Dental self-consciousness	Rural	6.69	1.838	0.294	0.858
	Urban	6.62	2.098	0.264	
Total PIDAQ score	Rural	28.28	6.017	0.964	0.288
	Urban	27.05	5.446	0.686	

* correlation is significant at the 0.05 level.** correlation is significant at the 0.01 level.***correlation is significant at the 0.001 level.

There are some methodological limitations inherent in this study which should be considered when generalizing findings. Firstly, the age of participants in both the experimental and control groups was low compared to a random sample of the general population. It is feasible that as people age beyond the age boundaries within this study, they will become differentially sensitive to differences of appearance due to craniofacial anomalies. A second limitation lies in the nature of the comparison group. As university students and employees, it is feasible that they are not directly equivalent to the patient group in cognitive and social domains. However, there is no *a priori* reason to assume that these differences would be related to subjective feelings or coping around appearance, and as such, the use of this comparison sample is justified. The comparison between rural and urban samples was not significant. This may, as described, reflect a genuine lack of difference. However, it is also worth reflecting on the categorization of these groups. For pragmatic reasons, current residence was used as the variable to code participants as ‘urban’ or ‘rural.’ What remains to be investigated in further work is the degree of stability of these categories. It may well be that those in the ‘urban’ category have been previously socialized in rural areas during formative periods of their development, and vice versa for those in the ‘rural’ category. If this were the case, differences between our groups would be less apparent than might be otherwise expected. In terms of the study instruments, both PIDAQ and DAS are robust measures. However, it is possible that there could be aspects of appearance sensitivity which are not identified in these measures.

## Conclusions

1. There is a significant psychological impact of altered facial and dental appearance in patients with craniofacial anomalies.
2. There was no significant effect of gender on the psychological impact of facial appearance in patients; however, significant negative psychological impact of dental appearance was seen in female patients.
3. There was no effect of locality (rural/urban) on the psychological impact of facial and dental appearance.
